# Input correlations impede suppression of chaos and learning in balanced firing-rate networks

**DOI:** 10.1371/journal.pcbi.1010590

**Published:** 2022-12-05

**Authors:** Rainer Engelken, Alessandro Ingrosso, Ramin Khajeh, Sven Goedeke, L. F. Abbott

**Affiliations:** 1 Zuckerman Mind, Brain, Behavior Institute, Columbia University, New York, New York, United States of America; 2 The Abdus Salam International Centre for Theoretical Physics, Trieste, Italy; 3 Neural Network Dynamics and Computation, Institute of Genetics, University of Bonn, Bonn, Germany; UCSD: University of California San Diego, UNITED STATES

## Abstract

Neural circuits exhibit complex activity patterns, both spontaneously and evoked by external stimuli. Information encoding and learning in neural circuits depend on how well time-varying stimuli can control spontaneous network activity. We show that in firing-rate networks in the balanced state, external control of recurrent dynamics, i.e., the suppression of internally-generated chaotic variability, strongly depends on correlations in the input. A distinctive feature of balanced networks is that, because common external input is dynamically canceled by recurrent feedback, it is far more difficult to suppress chaos with common input into each neuron than through independent input. To study this phenomenon, we develop a non-stationary dynamic mean-field theory for driven networks. The theory explains how the activity statistics and the largest Lyapunov exponent depend on the frequency and amplitude of the input, recurrent coupling strength, and network size, for both common and independent input. We further show that uncorrelated inputs facilitate learning in balanced networks.

## Author summary

Information in the brain is processed by a deeply-layered structure of local recurrent neural circuits. Recurrent neural networks often exhibit spontaneous irregular activity patterns that arise generically through the disordered interactions between neurons. Understanding under which conditions one circuit can control the activity patterns in another circuit and suppress spontaneous, chaotic fluctuations is crucial to unravel information flow and learning input-output tasks.

Here we find that when different neurons receive identical input, a larger input modulation amplitude is necessary to suppress chaos and facilitate learning in balanced firing-rate networks compared to different neurons receiving distinct input. This counterintuitive behavior is explained by a dynamic cancellation of common external input by recurrent currents—a feature previously described in balanced networks of binary neurons. We systematically study the scaling of this effect with different network parameters, describe high- and low-frequency limits analytically, and develop a novel non-stationary dynamic mean-field theory that predicts when chaos gets suppressed by correlated time-dependent input. Finally, we investigate the implications for learning in balanced firing-rate networks.

## Introduction

Neural circuits are highly interconnected, which generates complex activity dynamics both spontaneously and in response to incoming stimuli. Identifying mechanisms by which time-varying stimuli can control circuit dynamics is important for understanding information transmission, learning reliable input-output functions, and designing optogenetic stimulation protocols.

Recurrent neural networks provide a framework for understanding the interaction between external input and internally-generated dynamics. These networks can exhibit rich chaotic dynamics in the absence of external input [1]. External input can suppress chaotic dynamics, thus controlling the internal state of the network [2–4]. Such control of the recurrent dynamics appears necessary for reliable task learning [5–8].

Fundamental features of biological neural network dynamics include operation in continuous time, nonnegative firing rates and segregation of excitation and inhibition. Here we address input-driven network dynamics that adhere to these biological constraints. Excitation and inhibition in most biological circuits are conveyed by separate sets of neurons with a predominance of recurrent inhibitory feedback, a property known as ‘inhibition dominance’ [9–12]. Moreover, neurons in local populations receive time-dependent input that is correlated across neurons and can trigger a time-dependent population response. It is important to investigate how such biological features shape network dynamics, response to external inputs, and learning.

A class of recurrent network models originally proposed to explain the origins of asynchronous irregular activity is termed ‘balanced’ [13, 14]. In these networks, large excitatory inputs are dynamically canceled by strong recurrent inhibitory feedback. Firing-rate networks in the balanced state can exhibit chaotic activity fluctuations [15, 16], giving rise to complex activity patterns. How the dynamic cancellation described in balanced networks of binary neurons [13, 14] extends to firing-rate models and how it affects the suppression of chaotic dynamics has not yet been addressed. Previous dynamic mean-field theory (DMFT) approaches to input-driven rate networks assumed that the mean of the external input across neurons does not depend on time, which facilitates DMFT [2–4].

It remains unclear how external input should be structured to suppress chaos and control the network state effectively in rate networks satisfying biological constraints. To address this gap, we study stimulus-induced suppression of chaos in balanced rate networks with two types of time-dependent external input. Specifically, we study time-dependent input that is either identical across network neurons (referred to as common input) or that varies independently between the neurons (referred to as independent input).

We show that much larger input modulations are necessary to suppress chaos in networks that are driven by common input, because common input is canceled by strong recurrent inhibition in balanced networks. Conventional DMFT methods [1, 4, 15, 16] are not adequate to fully capture the effects of time-varying common input. Therefore, we developed a DMFT that is non-stationary, meaning that the order parameters can explicitly depend on time. This novel technique accurately captures the time-dependent mean and variance, the two-time autocorrelation function and the largest Lyapunov exponent of input-driven network dynamics. Specifically, we calculate the smallest input modulation amplitude required to suppress chaos, referred to as the critical input amplitude. Using both theory and simulations, we examine differences in the effect of common and independent input across a wide range of frequencies of the sinusoidal input modulation, weight heterogeneity and network sizes. We also provide approximations at low and high input frequencies. All the theoretical results match those from network simulations, provided the networks are sufficiently large.

Our findings have important implications for learning in balanced models and for fitting rate networks that obey biological constraints to neural data. We quantify how successful learning performance requires chaos suppression. As a result of residual chaotic fluctuations, common input that is used to suppress chaos during learning in a number of schemes [5–8] meets with limited success in balanced networks unless it has a very large amplitude. We show how the use of independent input resolves this problem.

## Results

### Chaos suppression with common vs independent input

We study how suppression of chaos depends on input correlations in balanced rate networks with time-dependent external input. For simplicity, we begin our analysis by studying a single inhibition-dominated population, where the recurrent inhibitory feedback dynamically balances a positive external input rather than recurrent excitation. The excitatory-inhibitory case is considered in a later section. Thus, we study a network of *N* nonlinear rate units (’neurons’) with dimensionless synaptic currents *h*_*i*_ and firing rates *ϕ*(*h*_*i*_) that obey

$$\begin{array}{c} \tau \frac{dh_{i}}{dt}=-h_{i}+\sum_{j=1}^{N}J_{ij}\phi (h_{j})+\sqrt{N}I_{0}+\delta I_{i}\left(t\right)\;, \end{array}$$ (1)

with i.i.d. Gaussian-distributed random couplings $J_{ij}∼N\left(-J_{0}/\sqrt{N},g^{2}/N\right)$, where the gain parameter *g* controls the weight heterogeneity of the network. The transfer function *ϕ* is set to a threshold-linear function *ϕ*(*x*) = max(*x*, 0). The $1/\sqrt{N}$ scaling of the negative mean coupling results in strongly negative recurrent feedback that dynamically cancels the constant input term $\sqrt{N}I_{0}$. In addition to this constant positive term, the external input contains a time-dependent component *δI*_*i*_(*t*).

**Fig 1:**
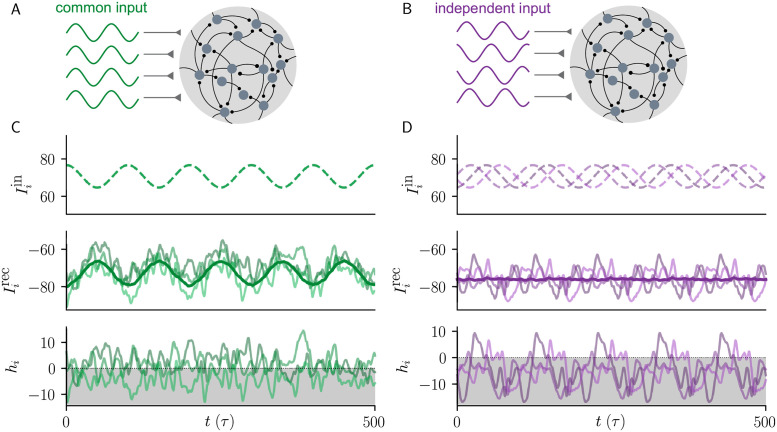
Suppression of chaos in balanced networks with common vs independent input. **A)** Common input: External input $I_{i}^{\text{in}}\left(t\right)=\sqrt{N}I_{0}+\delta I\left(t\right)$ consist of a positive static input and a sinusoidally time-varying input with identical phase across neurons. **B)** Independent input: External input $I_{i}^{\text{in}}\left(t\right)=\sqrt{N}I_{0}+\delta I_{i}\left(t\right)$ consist of a positive static input and a sinusoidally time-varying input with a random phase for each neuron. **C)** External inputs (top), recurrent feedback $I_{i}^{\text{rec}}=\sum_{j}J_{ij}\phi (h_{j})$ and their population average (thick line) (middle), and synaptic currents (bottom) for three example neurons. Recurrent feedback has a strong time-varying component that is anti-correlated with the external input, resulting in cancellation. **D)** Same as in **C**, but for independent input. Here, no cancellation occurs and the network is entrained into a forced limit cycle. Throughout this work, green (violet) refers to common (independent) input. Model parameters: *N* = 5000, *g* = 2, *f* = 0.01/*τ*, *I*_0_ = *J*_0_ = 1, *I*_1_ = 6.

Throughout, we distinguish between two types of time-dependent inputs, ‘common’ vs ‘independent’. In both cases, the time-dependence is sinusoidal, but for common input, *δI*_*i*_(*t*) = *δI*(*t*) = *I*_1_ sin(2*πft*), which is identical across network neurons (Fig 1A). For independent input, *δI*_*i*_(*t*) = *I*_1_ sin(2*πft* + *θ*_*i*_) has an independent random phase for each neuron (Fig 1B), with phase *θ*_*i*_ drawn independently from a uniform distribution between 0 and 2*π*. We assume that *N* is large enough or the phases are appropriate so that we can take the average of *δI*_*i*_(*t*) across the population to be zero in the independent case. The amplitude of *δI*_*i*_(*t*) is denoted by *I*_1_, and *f* is the input frequency. We will investigate in the following how large *I*_1_ has to be, and how it has to scale with network size, in order to control the dynamics of recurrent networks and suppress chaotic fluctuations for the two input types. Therefore, we do not a assume priori any particular scaling of *I*_1_ with network size in Eq 1.

For firing-rate networks in the balanced state, suppression of chaos strongly depends on the correlations of the input (Fig 1). One might expect that driving all neurons with a common input would be an effective way to suppress chaos, but input that is shared across neurons recruits strong recurrent inhibitory feedback that is anti-correlated with the common input (Fig 1C). This means that the time-varying external input is dynamically canceled by recurrent feedback, leaving behind only a small fraction of the time-dependent common input for chaos suppression. In contrast, for independent input, which is randomly phase-offset across neurons, no such cancellation occurs (Fig 1D), and thus weaker external input is required to suppress chaotic fluctuations in the network.

To understand how this discrepancy arises in the model, it is useful to rewrite Eq 1 by decomposing $h_{i}=m+{\tilde{h}}_{i}$ into a mean component *m* and residual fluctuations ${\tilde{h}}_{i}$. We decompose $J_{ij}=-J_{0}/\sqrt{N}+{\tilde{J}}_{ij}$, where the entries of ${\tilde{J}}_{ij}$ are Gaussian with variance *g*^2^/*N* and mean zero. For common input, this results in

$$\begin{array}{ccc} \tau \frac{dm}{dt} & =-m-\sqrt{N}J_{0}\nu \left(t\right)+\sqrt{N}I_{0}+\delta I\left(t\right)\;, & \left(2\text{a}\right)\\ \tau \frac{d{\tilde{h}}_{i}}{dt} & =-{\tilde{h}}_{i}+\sum_{j=1}^{N}{\tilde{J}}_{ij}\phi (m+{\tilde{h}}_{j})\; & \left(2\text{b}\right) \end{array}$$

with mean population firing rate $\nu \left(t\right)=\frac{1}{N}\sum_{i}\;\phi \left(h_{i}\left(t\right)\right).$ Here *δI*(*t*) directly enters the expression for *m*, because it is identical across all neurons. It thus directly impacts *ν*(*t*) and recruits, through the negative recurrent mean coupling $-J_{0}/\sqrt{N}$, strong recurrent feedback $-\sqrt{N}J_{0}\nu$ that is anti-correlated with the input and cancels most of both the positive static input and the time-dependent common component of the total external input. Solving Eq 2a for the population firing rate *ν*(*t*) yields:

$$\begin{array}{c} \;\nu \left(t\right)=\frac{I_{0}}{J_{0}}+\frac{1}{J_{0}\sqrt{N}}(\delta I\left(t\right)-\tau \frac{dm}{dt}-m)\;. \end{array}$$ (3)

In the absence of time-dependent input, this equation is commonly referred to as the ‘balance equation’ [14–16]. Note that the impact of *δI*(*t*) on the population firing rate is reduced by a factor of $1/\sqrt{N}$.

With independent input, Eq 1 can be written as

$$\begin{array}{ccc} \tau \frac{dm}{dt} & =-m-\sqrt{N}J_{0}\nu \left(t\right)+\sqrt{N}I_{0}\;, & \left(4\text{a}\right)\\ \tau \frac{d{\tilde{h}}_{i}}{dt} & =-{\tilde{h}}_{i}+\sum_{j=1}^{N}{\tilde{J}}_{ij}\phi (m+{\tilde{h}}_{j})+\delta I_{i}\left(t\right)\;. & \left(4\text{b}\right) \end{array}$$

In this case, *δI*_*i*_(*t*) enters the equation for the fluctuations ${\tilde{h}}_{i}$. Thus, the strong recurrent feedback only cancels the positive static input term, $\sqrt{N}I_{0}$. Chaos, in this case, is suppressed through the influence of *δI*_*i*_(*t*) on the fluctuations ${\tilde{h}}_{i}$, similar to what happens in the case of random non-balanced networks [2–4].

**Fig 2:**
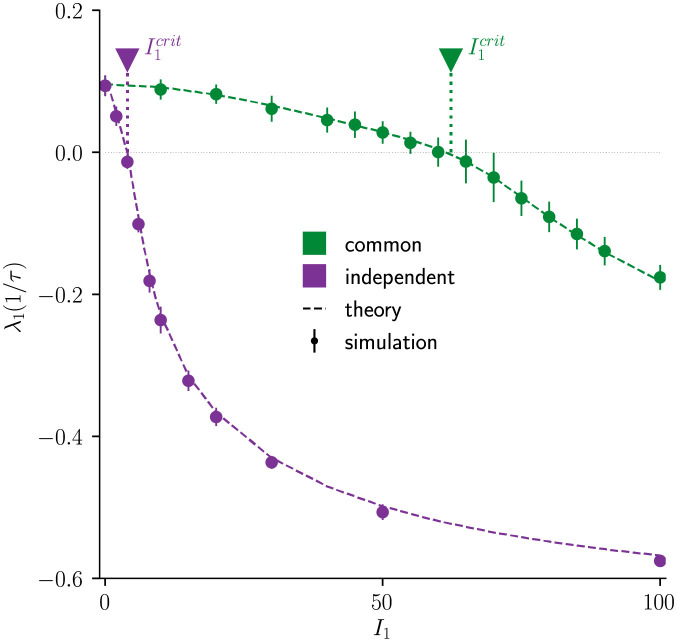
Largest Lyapunov exponent shows different chaos suppression for common vs independent input. Largest Lyapunov exponent λ_1_ as a function of input modulation amplitude *I*_1_ for common (green) and independent (violet) input. $I_{1}^{\text{crit}}$ are the zero-crossings of λ_1_ and thus the minimum *I*_1_ required to suppress chaotic dynamics. With common input, λ_1_ crosses zero at a much larger *I*_1_. Dots with error bars are numerical simulations, dashed lines are largest Lyapunov exponents computed by dynamic mean-field theory (DMFT). Error bars indicate ±2 std across 10 network realizations. Model parameters: *N* = 5000, *g* = 2, *f* = 0.2/*τ*, *I*_0_ = *J*_0_ = 1.

We quantify chaos in the network dynamics by the largest Lyapunov exponent λ_1_. This quantity measures the average exponential rate of divergence or convergence of nearby network states [17] and is positive if the network dynamics is chaotic. We computed λ_1_ analytically using non-stationary DMFT (Materials and Methods) and confirmed the results by simulations of the full network dynamics. For both common and independent input, λ_1_ is a decreasing function of the input amplitude *I*_1_ and crosses zero at a critical input amplitude $I_{1}^{\text{crit}}$ (Fig 2). With common input, a much larger value of *I*_1_ is required for λ_1_ to become negative and thus for chaos suppression.

### Dependence on network parameters

**Fig 3:**
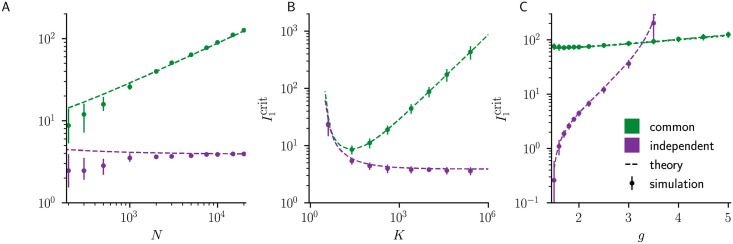
Difference in chaos suppression increases with network size, tightness of balance, and near the transition to chaos. **A)** Dependence of $I_{1}^{\text{crit}}$ on network size *N*. With common input, $I_{1}^{\text{crit}}\propto \sqrt{N}$ for large *N*, but is constant for independent input. Error bars indicate interquartile range around the median. **B)** Dependence of $I_{1}^{\text{crit}}$ on ‘tightness of balance’ parameter *K*, which scales both *I*_0_ and *J*_0_. Results for large *K* are the same as in A but for small *K*, the network is no longer in the balanced regime, and results for common and independent input become similar. Error bars indicate ±2 std. **C)** Dependence of $I_{1}^{\text{crit}}$ on gain parameter *g* for low input frequency *f*. Close to $g_{\text{crit}}$, an arbitrarily small independent input can suppress chaos; this is not the case with common input. The quasi-static approximation (dotted) and DMFT (dashed) results coincide. Error bars indicate ±2 std. Model parameters: *I*_0_ = *J*_0_ = 1 in **A** and **C**; *g* = 2, *f* = 0.2/*τ* in **A** and **B**; $I_{0}=J_{0}=\sqrt{K/N}$, in **B**; *f* = 0.01/*τ* in **C**, *N* = 5000 in **B** and **C**.

Next, we explore how $I_{1}^{\text{crit}}$ varies between networks driven by common and independent input. As suggested by Eqs 2 and 4, the discrepancy between common and independent input grows with network size *N*. For common input, $I_{1}^{\text{crit}}$ is proportional to $\sqrt{N}$ for large *N*, while it saturates as a function of *N* for independent input (Fig 3A). Thus, an ever-increasing *I*_1_ is required to suppress chaotic activity in larger networks that are driven by common input. Note that the agreement between theory and simulations is good for large *N* (Fig 3A).

In balanced networks, the network size *N* acts as a scale factor for the mean of the coupling weights and the magnitude of the constant external input (Eq 1). Mean-field theory describes the limit when the number of neurons goes to infinity, but it still contains *N* as a parameter multiplying these terms (Materials and Methods). To separate these two different aspects, we introduce a ‘tightness of balance’ parameter *K* by scaling both *J*_0_ and *I*_0_ with a factor $\sqrt{K/N}$. This removes the *N*-dependence in the DMFT equations. *K* mimics the effect of changing the number of synapses per neuron on the mean current *m* in a random sparse network [13, 15, 16]. This allows us to vary the ‘tightness of balance’ [18, 19], while still studying networks with large enough *N* so that mean-field theory applies (Fig 3B).

We observe that for sufficiently large *K*, the dependence on *K* matches that on *N* in the unscaled model (Fig 3A): for common input, $I_{1}^{\text{crit}}$ is proportional to $\sqrt{K}$ and for independent input, $I_{1}^{\text{crit}}$ is independent of *K*. However, the qualitative difference between independent and common input vanishes for small values of *K* because the network is no longer in the regime of the balanced state. In the balanced regime, we expect the qualitative difference to be largely independent of the transfer function *ϕ*. For non-balanced networks with zero mean couplings and tanh transfer function, we also found no qualitative difference between common and independent input (Materials and Methods).

In balanced networks, the difference in $I_{1}^{\text{crit}}$ for common and independent input increases for decreasing gain parameter *g*. With independent input, $I_{1}^{\text{crit}}$ becomes arbitrarily small as *g* approaches $g_{\text{crit}}=\sqrt{2}$ (Fig 3C). At this critical gain parameter, the network with constant external input transitions from a fixed point to chaos [16]. At low frequency, $I_{1}^{\text{crit}}$ remains of order $\sqrt{N}$ even near $g_{\text{crit}}$ for common input (Fig 3C). We note that if *N* is fixed but *g* is increased to large values, $I_{1}^{\text{crit}}$ for independent input becomes larger than $I_{1}^{\text{crit}}$ for common input. The reason is that the variance of the synaptic currents *h*_*i*_(*t*) grows faster for independent input than for common input as the network approaches a global instability where the dynamics diverges.

### Mechanism of chaos suppression for slowly varying common input

**Fig 4:**
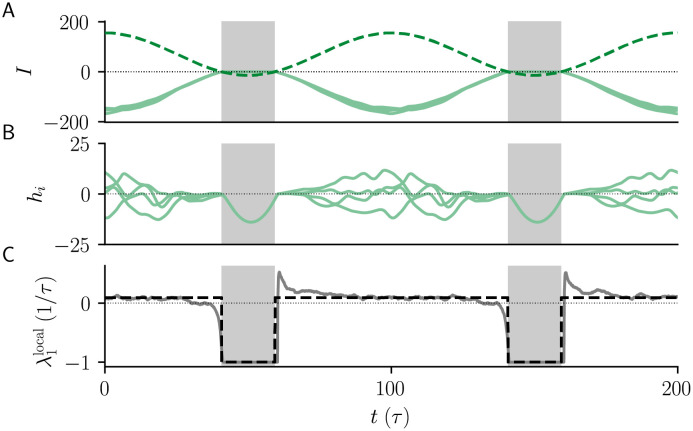
Mechanism of chaos suppression with slowly varying common input. **A)** External input $I_{i}^{\text{in}}\left(t\right)=\sqrt{N}I_{0}+\delta I_{i}\left(t\right)$ (dashed) and recurrent input $I_{i}^{\text{rec}}\left(t\right)=\sum_{j}\;J_{ij}\phi (h_{j}\left(t\right))$ (solid) for three example neurons. **B)** Synaptic currents *h*_*i*_ for four example neurons. **C)** Local Lyapunov exponent from network simulation, which reflects the local exponential growth rates between nearby trajectories (solid), and Lyapunov exponent from stationary DMFT (dashed) used in quasi-static approximation. When $I_{1}>\sqrt{N}I_{0}$, external input periodically becomes negative and silences the recurrent activity (gray bars). During these silent episodes, the network is no longer chaotic and $\lambda_{1}^{\text{local}}=-1/\tau$. When the input is positive, dynamics remains chaotic and $\lambda_{1}^{\text{local}}>0$ on average. Model parameters: *N* = 5000, *g* = 2, *f* = 0.01/*τ*, *I*_0_ = *J*_0_ = 1.

An intuitive picture of chaos suppression by common sinusoidal input can be provided in the limit of low frequency, where the input varies more slowly than the intrinsic network fluctuations. We call this limit the quasi-static approximation. In this limit, when *I*_1_ exceeds the static external input $\sqrt{N}I_{0}$, recurrent activity is periodically silenced (Fig 4A and 4B). During these silent episodes, all neurons intermittently approach the locally stable dynamics $h_{i}\left(t\right)=\sqrt{N}I_{0}+\delta I\left(t\right)<0$. On the other hand, when $g>g_{\text{crit}}$ any positive external input result in chaos [16]. Thus, in a quasi-static approximation, λ_1_ is given by averaging the local Lyapunov exponent $\lambda_{1}^{\text{local}}$ across the silent and chaotic episodes, weighted by their respective durations (Fig 4C; Materials and Methods; $\lambda_{1}^{\text{local}}$ is approximated using DMFT). During the silent episodes, $\lambda_{1}^{\text{local}}=-1/\tau$. In the chaotic episodes, $\lambda_{1}^{\text{local}}>0$ depends on how far the network is from the transition to chaos, i.e., on the gain parameter *g*. As a result, $I_{1}^{\text{crit}}$ is determined by the duration of the silent episodes that is required to compensate the remaining transiently chaotic dynamics, and it grows monotonically with *g* (Fig 3C) because longer silent episodes are necessary to compensate for the stronger chaotic activity.

This quasi-static perspective might suggest that chaos can only be suppressed by common input when $I_{1}>\sqrt{N}I_{0}$, so that there exist ‘silent episodes’ where the total input into neurons $\sqrt{N}I_{0}+\delta I\left(t\right)$ is intermittently negative and firing rates are zero. That finding is however only correct in the limit *f* → 0. For finite input frequency, chaos can be suppressed by a common input even when the external input is always positive.

### Frequency-dependent chaos suppression

**Fig 5:**
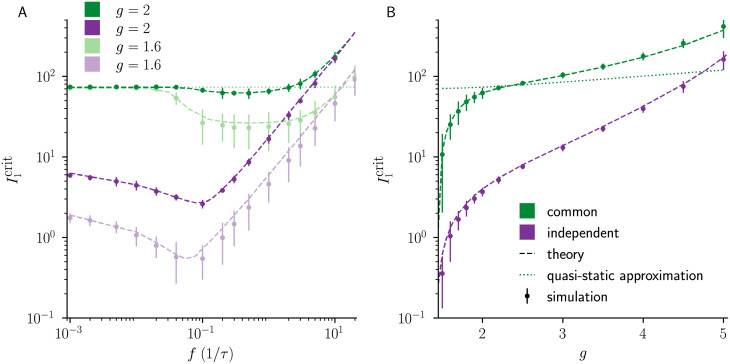
Dynamic mean-field theory captures frequency-dependent effects on the suppression of chaos. **A)** $I_{1}^{crit}$ as a function of input frequency *f* (*g* = 1.6 light color, *g* = 2 dark color). $I_{1}^{\text{crit}}$ has a minimum that is captured by the non-stationary DMFT (dashed green line) but not by the quasi-static approximation (dotted green line), which does not depend on frequency *f*. At high *f*, the low-pass filter effect of the leak term attenuates the external input modulation for both cases, thus resulting in a linearly increasing $I_{1}^{\text{crit}}$. **B)** Dependence of $I_{1}^{\text{crit}}$ on the gain parameter *g* for high input frequency (*f* = 0.2/*τ*), showing a monotonic increase. The non-stationary DMFT results are in good agreement with numerical simulations. For comparison, we include the result of the quasi-static approximation (dotted green line), which shows a more gradual dependence on *g* and applies only at low frequencies (see Fig 3). Error bars indicate ±2 std. Model parameters: *N* = 5000, *g* = 2, *f* = 0.2/*τ*, *I*_0_ = *J*_0_ = 1.

We next explore the effects of the frequency of the sinusoidal input on $I_{1}^{\text{crit}}$. For both common and independent input, we observe a ‘resonant frequency’ at which the input is most effective at suppressing chaos (Fig 5A). For common input, at low frequency, $I_{1}^{\text{crit}}$ is insensitive to the frequency and is thus well approximated by the quasi-static approximation described above. However, for increasing frequencies, $I_{1}^{\text{crit}}$ exhibits a minimum in *f*, which can only be captured by non-stationary DMFT (Materials and Methods). For both common and independent input, when the frequency is high, low-pass filtering originating from the leak term in Eq 1 attenuates the effective input modulation amplitude by a factor of $1/\sqrt{1+{\left(2\pi f\tau \right)}^{2}}$. As a result, a stronger input modulation amplitude is required to compensate the effect of this attenuation, and $I_{1}^{\text{crit}}$ exhibits a linear increase with *f* (Fig 5A). We find that also for independent input, $I_{1}^{\text{crit}}$ exhibits a minimum in *f*, an effect previously reported for networks that have zero mean couplings, *J*_0_ = 0, and a sigmoidal transfer function [3]. The resonant frequency originates from a superposition of two distinct effects: for increasing frequency, the input decorrelates subsequent Jacobians, which makes the network less chaotic and thus leads to smaller $I_{1}^{\text{crit}}$[4, 17]. For ever-increasing frequencies, however, the input is increasingly attenuated by the filtering effect of the leak term, which overcompensates the decorrelation effect for large *f*.

We also examined the effect of the coupling gain *g* on the critical input amplitude $I_{1}^{\text{crit}}$. For low input frequencies, a finite value $I_{1}^{\text{crit}}$ occurred near the onset of chaos at $g=g_{\text{crit}}$ (Fig 3C). At a higher frequency, *f* = 0.2/*τ*, this is no longer the case (Fig 5B), which is captured by the non-stationary DMFT. Close to $g_{\text{crit}}$, the critical input amplitude is small for both common and independent input.

Collectively, these results demonstrate that a larger input amplitude is necessary to suppress chaotic dynamics when balanced networks are driven by common, as opposed to independent input, and that non-stationary DMFT successfully captures this effect in large networks.

### Chaos suppression in balanced two-population E-I network

**Fig 6:**
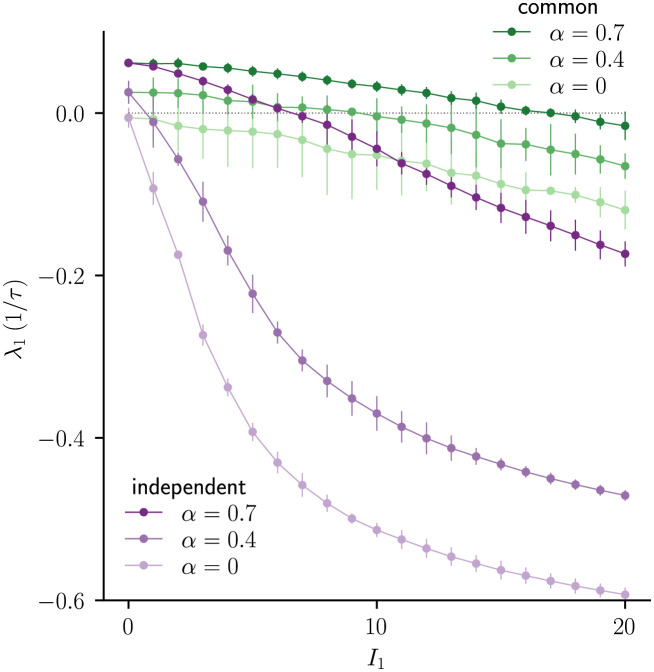
Difference in chaos suppression in sparsely-connected E-I network. λ_1_ as a function of *I*_1_ for common and independent inputs, showing a monotonic decrease with *I*_1_ and a larger zero-crossing for common input. This result is qualitatively similar to that obtained in the single population network with negative mean coupling (Fig 2). Error bars indicate ±2 std, lines are a guide for the eye. Increasing the excitatory efficacy *α* increases λ_1_ for both common and independent input (*α* ∈ {0, 0.5, 0.7}). Model parameters (parameters defined as in [16] for constant input and *W*_*I*1_ and *W*_*E*1_ are the modulation amplitudes of the input to the excitatory and inhibitory population): *N*_*E*_ = *N*_*I*_ = 3500, *K* = 700, *g* = 1.6, $J_{EE}=g\alpha /\sqrt{K}$, $J_{EI}=-1.11g/\sqrt{K}$, $J_{IE}=g\alpha /\sqrt{K}$, $J_{II}=-g/\sqrt{K}$, $W_{E}=g\alpha \sqrt{K}$, $W_{I}=0.44g\sqrt{K}$, *W*_*E*1_ = *gαI*_1_, *W*_*I*1_ = 0.44*gI*_1_, *f* = 0.2/*τ*.

The effect that we report for a fully-connected random network of neurons with negative mean coupling extends to a sparsely-connected two population excitatory-inhibitory network in the balanced state. We calculate the largest Lyapunov exponent λ_1_ as a function of input amplitude *I*_1_ in network simulations and find that, consistent with our earlier observations, a much stronger input is required for common input to reduce λ_1_ to zero and consequently suppress the chaotic activity (Fig 6).

Because of the additional parameters, two population excitatory-inhibitory network can exhibit more complex behaviors [13, 15, 16, 20]. Here we consider a one-dimensional parametrization by the excitatory efficacy *α*, a parameter that multiplies all excitatory couplings as described in [16]. We observe numerically that increasing the excitatory efficacy *α* increases λ_1_ for both common and independent input (Fig 6). We leave a detailed theoretical analysis of two population excitatory-inhibitory network, including the effect of different time constants that is known to affect chaos [15], for future work.

### Training balanced networks with common vs independent input

**Fig 7:**
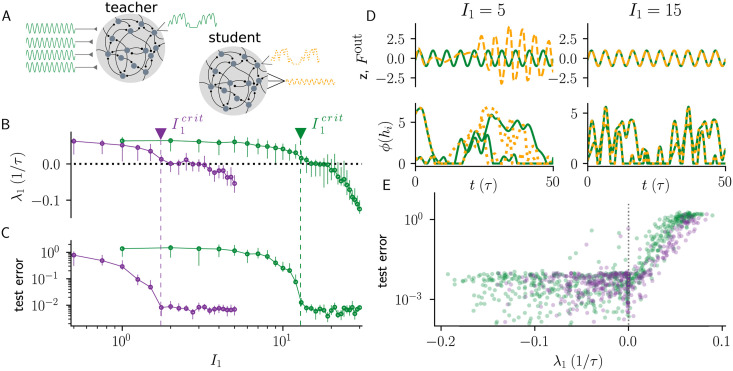
Common input impedes learning in balanced networks. **A)** Schematic of the training setup. A ‘student network’ (S) is trained to autonomously generate the output $F^{\text{out}}\left(t\right)=\sin(2\pi ft)$, by matching its recurrent inputs to those of a driven ‘teacher network’, whose weights are not changed during training. **B)** λ_1_ in the teacher network as a function of *I*_1_. **C)** Test error in the student network as a function of *I*_1_. Critical input amplitude $I_{1}^{\text{crit}}$ is indicated by vertical dashed lines. Consistent with the difference in $I_{1}^{\text{crit}}$, the teacher networks driven with common input require a larger *I*_1_ to achieve small test errors in the student network. Error bars indicate interquartile range around the median. **D)** Top: Target output $F^{\text{out}}$ (green) and actual output *z* (dashed orange) for two input amplitudes *I*_1_ ∈ {5, 15}. Bottom: Firing rate *ϕ*(*h*_*i*_) for two example neurons in teacher network with common input (green full line) and student network (orange dotted line) for two input amplitudes. **E)** Scatter plot of test error as a function of λ_1_ for each network realization in **A** and **B**, with both common and independent input. When chaos in the teacher network is not suppressed (λ_1_ > 0), test error is high. Training is successful (small test error) when targets are strong enough to suppress chaos in the teacher network. Training is terminated when error reaches below 10^−2^. Model parameters: *N* = 500, *g* = 2, *I*_0_ = *J*_0_ = 1, *ϕ*(*x*) = max(*x*, 0) in both teacher and student networks; *f* = 0.2/*τ* in the teacher network inputs and target $F^{\text{out}}$.

Our results on the impact of common versus independent input have important implications for learning in recurrent networks. To address this issue, we considered a target-based approach for task learning, called full-FORCE [6, 8]. In this learning procedure, a ‘student network’ (S) learns a task by matching its recurrent inputs to those of a ‘teacher network’ (Fig 7A). The teacher network is randomly connected and driven by the desired output to generate the target currents. The synaptic coupling matrix of the student network is then trained by an online learning algorithm to autonomously generate the desired output (Materials and Methods).

We consider a case in which the task of the student network is to autonomously generate the target output $F^{\text{out}}\left(t\right)=\sin\left(2\pi ft\right)$. In the standard student-teacher network setup [6, 8], an input proportional to this desired output, *δI*_*i*_(*t*) = *I*_1_ sin(2*πft*), would be injected into each unit of the teacher network. However, in a balanced network, as we have shown, this is not an efficient way to suppress chaos within the teacher network; an input of the form *I*_1_ sin(2*πft* + *θ*_*i*_) with varying phases will be far more effective.

We examine learning using teacher networks set up according to Eq 1 with each neuron *i* driven by *δI*_*i*_(*t*) = *I*_1_ sin(2*πft* + *θ*_*i*_). We systematically studied the influence of common input (same *θ*_*i*_ across the teacher network) and independent input (random *θ*_*i*_ across the teacher network) on learning performance in the student network. In both cases, test error drops when chaos is suppressed in the teacher network, as signaled by the zero-crossing of λ_1_ (Fig 7B), but a much larger value of *I*_1_ is required to obtain the same test error with common input than with independent input.

In Fig 7D, we show examples of firing rates in both the teacher and student network, for two different values of input amplitude *I*_1_ during testing. The readout *z*(*t*) = ∑_*i*_ *w*_*i*_ *ϕ*(*h*_*i*_(*t*)) is also shown. When the teacher network dynamics is chaotic, the readout *z*(*t*) quickly deviates from the target output. Crucially, chaos suppression in the teacher network is not induced by intermittent silencing of the whole teacher network: a reliable readout *z* can be produced when the external input $\sqrt{N}I_{0}+\delta I\left(t\right)>0$ at all times.

The impact of chaos on task performance is more striking when the test error is plotted against λ_1_ for individual network realizations (Fig 7D), demonstrating that trained networks with small test error correspond to ones where the time-varying inputs suppresses chaos in the teacher network. Interestingly, in some cases, the student network can learn to approximately reproduce the prescribed dynamics even when the teacher network is slightly in the chaotic regime (small but positive λ_1_).

### Firing rates and autocorrelations of balanced networks with common and independent input

**Fig 8:**
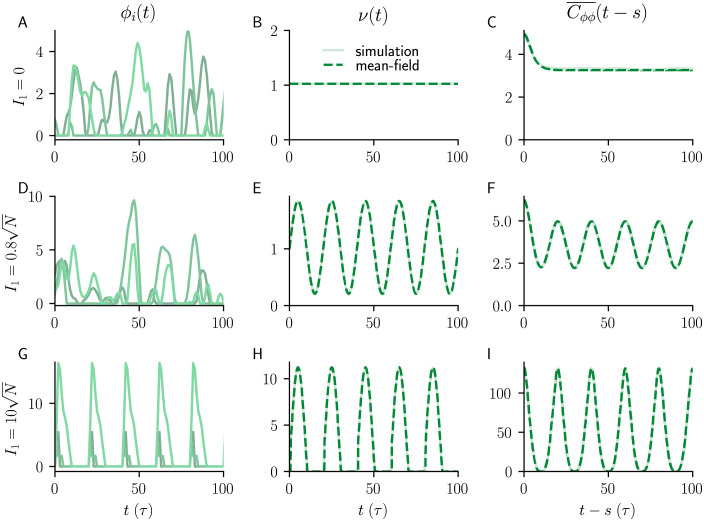
Activity, population firing rate and autocorrelations of balanced networks with common input. **A)** Firing rates *ϕ*_*i*_(*t*) = *ϕ*(*h*_*i*_(*t*)) of three example units. **B)** Mean population firing rate *ν*(*t*). **C)** Time-averaged two-time autocorrelation function (Eq 5) as a function of time difference with no external input (*I*_1_ = 0). **D-F)** Same as **A-C** but for input amplitude $I_{1}=0.8\sqrt{N}\approx 56.5$; activity remains chaotic. **G-I** Same as **A-C** but for stronger input ($I_{1}=10\sqrt{N}\approx 707.1$); activity is entrained by the external input and is no longer chaotic. Dashed lines (middle and right columns) are results of non-stationary DMFT, full lines are median across 10 network realizations. Model parameters: *N* = 5000, *g* = 2, *f* = 0.05/*τ*, *I*_0_ = *J*_0_ = 1.

The non-stationary DMFT also accurately describes the mean population rate *ν*(*t*) and the two-time autocorrelation function of the residual fluctuations ${\tilde{h}}_{i}$ in the case of common input. In Fig 8, we compare non-stationary DMFT and numerical neural network simulations with common input in both the chaotic (cyclostationary) and stable (periodic) regimes. We consider the population-averaged autocorrelation function

$$\begin{array}{c} C_{\phi \phi }(t\prime +t,t\prime +s)=\langle \phi \left(h_{i}\left(t\prime +t\right)\right)\phi \left(h_{i}\left(t\prime +s\right)\right)\rangle \end{array}$$ (5)

of the fluctuating single neuron rates *ϕ*(*h*_*i*_), temporally averaged over *t*′ making $\bar{C_{\phi \phi }}\left(t-s\right)$ a function of the time difference *t* − *s*. Here the angular bracket represent either an population average or the stochastic average according to DMFT. Code for the non-stationary DMFT is made available here.

**Fig 9:**
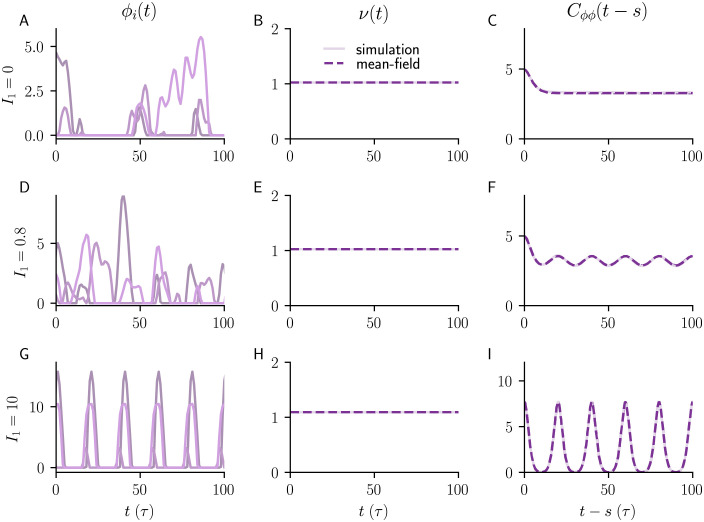
Activity, population firing rate and autocorrelations of balanced networks with independent input. **A)** Firing rates *ϕ*_*i*_(*t*) = *ϕ*(*h*_*i*_(*t*)) of three example units. **B)** Mean population firing rate *ν*(*t*). **C)** Autocorrelation function with no external input (*I*_1_ = 0). **D)-F)** Same as **A-C** but for input amplitude of *I*_1_ = 0.8; activity remains chaotic. **G)-I)** Same as **A-C** but for stronger input (*I*_1_ = 10); activity is fully controlled by the external input and is no longer chaotic. Dashed lines (middle and right columns) are results of stationary DMFT, full lines are median across 10 network realizations. Model parameters: *N* = 5000, *g* = 2, *f* = 0.05/*τ*, *I*_0_ = *J*_0_ = 1.

The excellent agreement also holds for independent input (Fig 9), extending previous results of stationary DMFT for driven networks [3] to balanced networks.

## Discussion

We investigated how correlations in the external input influence suppression of chaos and learning in balanced networks. Stronger input modulations are required to suppress chaos when the inputs are correlated across neurons. The discrepancy between common and independent input increases for large network size, deep in the balanced regime, and in the vicinity of the transition to chaos of the autonomous dynamics. We developed a non-stationary dynamic mean-field theory to explain the dynamical effects of time-varying input (Materials and Methods). Furthermore, we demonstrated that this discrepancy affects task learning in balanced networks.

Our study is relevant in light of recent advances in optogenetics that allow for time-dependent stimulation of a selected population of neurons. Theoretical models that distinguish between different network dynamic regimes are of interest for this purpose [10, 19, 21]. Our work addresses this question through the spatiotemporal structure of the feedforward input. One experimental prediction of our work is that, if cortical circuits are in the balanced state, time-varying stimulation that is common across neurons will not suppress response firing-rate variability of firing rates as effectively as independently time-varying stimulation.

For multi-layered networks, common feedforward input from one balanced neural population with asynchronous activity into another population results in a time-averaged current that is proportional to $\sqrt{N}$ [14] but its temporal fluctuations would be small and according to our theory unsuitable to suppress rate chaos. In contrast, if the driving population has synchronous rate fluctuations, the mean of the current fluctuations would be proportional to $\sqrt{N}$ and thus be suitable to suppress rate chaos. We conclude that common input in biological neural circuits is not able to control a downstream population state unless the driving population is in a synchronous state.

Previous studies on suppression of chaos in rate networks were limited to independent inputs in the form of stochastic [2, 4] and sinusoidal [3] time-dependent drive, but the networks were not balanced, and their connectivity had zero mean coupling. In these previous studies, the distribution of the inputs across neurons in the population is time-independent [2–4] and stationary DMFT was sufficient to describe the results. In contrast, the treatment of common input is only possible by the non-stationary dynamic mean-field approach introduced here.

The dynamic cancellation of time-varying input through recurrent inhibitory feedback has been previously studied in balanced networks with binary [14, 22, 23] and spiking neurons [24, 25]. Chaos in balanced firing-rate networks was studied previously [4, 15, 16, 20], but the dynamic cancellation of correlated input and its implications on chaos suppression in rate networks were not investigated, nor were the implications for learning. It would be interesting to investigate the influence of input correlations on chaos in alternative models of the balanced state [10, 21] and recurrent networks with low-rank structure [26–29].

The different underlying mechanisms of chaos suppression for common and independent input we report here are not specific to periodic input modulations and a threshold-linear transfer function, which we merely chose for the sake of simplicity and analytical tractability. Balanced rate networks driven by stochastic inputs, such as an Ornstein-Uhlenbeck (OU) process, exhibit a qualitatively similar discrepancy between common and independent inputs (Materials and Methods). In that case, common input corresponds to a situation where all neurons receive the same realization of the OU process, with the intensity of the noise serving as the input amplitude. Moreover, a similar qualitative difference between independent and common input is expected in spiking balanced networks with sufficiently slow synaptic dynamics [15].

The ability to control the dynamics of recurrent networks is closely linked to the problem of learning. A target-based approach to supervised learning in recurrent networks provides a convenient framework for studying the link between chaos and trainability. This is because in this approach, as opposed to backpropagation through time for example, learning-induced changes in connectivity are uncoupled from the dynamics: whether chaos is suppressed in the teacher network does not depend on synaptic changes in the student network. We found that the reduced impact of common input on chaos suppression is reflected in learning performance: when the targets fail to suppress chaos in the teacher network, target trajectories cannot be learned reliably and, as a result, the student network fails to learn the task. This is not only relevant for computational studies of training recurrent neural networks in the balanced state [6–8], but also for fitting recurrent network models to neural data [30, 31] when imposing biological constraints such as inhibition-dominance, non-negative firing rates and correlated external inputs.

Based on our analysis, we propose two strategies to overcome this problem. One strategy is to time-offset the time-varying input into the teacher network across neurons (as in independent input explored in this study) so that their population average is approximately zero. An alternative approach is to project the target through input weights with significant variability across neurons in the population. Both solutions avoid a large time-varying mean component in the external input that would otherwise be dynamically canceled by recurrent feedback. In sum, the uncovered discrepancy can help to harness the computational capabilities of balanced networks for learning stable trajectories.

## Materials and methods

We analyze the dynamics of Eq 1 with time-varying common or independent external input. For common input, we develop a novel non-stationary dynamic mean-field theory (DMFT) yielding time-dependent population firing rates, two-time autocorrelation functions of the activity fluctuations and the largest Lyapunov exponents. For independent input, we calculate autocorrelation functions and Lyapunov exponents using stationary DMFT [1, 4, 15, 16], extending previous work [3].

We consider a single population of neurons with negative mean coupling, with the dynamic equation (see Eq 1) that we repeat here for convenience,

$$\begin{array}{c} \tau \frac{dh_{i}}{dt}=-h_{i}+\sum_{j}J_{ij}\phi (h_{j})+\sqrt{N}I_{0}+\delta I_{i}\left(t\right)\;. \end{array}$$

As mentioned in the main text, we decompose $h_{i}=m+{\tilde{h}}_{i}$ and $J_{ij}=-J_{0}/\sqrt{N}+{\tilde{J}}_{ij}$, where the entries of ${\tilde{J}}_{ij}$ are i.i.d. Gaussian with variance *g*^2^/*N* and mean zero. For convenience, we include here the decompositions of Eq 1 for common input,

$$\begin{array}{ccc} \tau \frac{dm}{dt} & =-m-\sqrt{N}J_{0}\nu \left(t\right)+\sqrt{N}I_{0}+\delta I\left(t\right)\;, & \left(7\text{a}\right)\\ \tau \frac{d{\tilde{h}}_{i}}{dt} & =-{\tilde{h}}_{i}+\sum_{j}{\tilde{J}}_{ij}\phi (m+{\tilde{h}}_{j}) & \left(7\text{b}\right) \end{array}$$

and for independent input,

$$\begin{array}{ccc} \tau \frac{dm}{dt} & =-m-\sqrt{N}J_{0}\nu \left(t\right)+\sqrt{N}I_{0}\;, & \left(8\text{a}\right)\\ \tau \frac{d{\tilde{h}}_{i}}{dt} & =-{\tilde{h}}_{i}+\sum_{j}{\tilde{J}}_{ij}\phi (m+{\tilde{h}}_{j})+\delta I_{i}\left(t\right)\;. & \left(8\text{b}\right) \end{array}$$

with the network-averaged (population) firing rate $\nu \left(t\right)=\frac{1}{N}\sum_{i=1}^{N}\phi (m\left(t\right)+{\tilde{h}}_{i}\left(t\right))$.

### Common input

After developing a non-stationary DMFT for the dynamics given above with common input, we analyze the small and large frequency limits.

#### Non-stationary dynamic mean-field theory

In this section, we derive a non-stationary DMFT for common input starting from Eq 2. With time-dependent common input to all units, the mean component *m*(*t*) and the autocorrelations of the residual fluctuations ${\tilde{h}}_{i}\left(t\right)$ change over time. Therefore, the statistics of *h*_*i*_ are not stationary, in contrast to what is assumed in conventional DMFT approaches [1, 3, 4, 15, 16, 18, 32, 33].

The basic idea of DMFT is that for large *N*, the distribution of the recurrent input for different neurons becomes Gaussian and pairwise uncorrelated, according to the central limit theorem. To this end, we characterize the distribution of the residual fluctuations ${\tilde{h}}_{i}\left(t\right)$ by considering the (linear) stochastic dynamics

$$\begin{array}{c} \tau \frac{d\tilde{h}}{dt}=-\tilde{h}+\eta \left(t\right)\;, \end{array}$$ (9)

where *η*(*t*) is a Gaussian process with mean 〈*η*(*t*)〉 = 0 and autocorrelation

$$\begin{array}{c} q\left(t,s\right)=\left\langle \eta \left(t\right)\eta \left(s\right)\right\rangle =g^{2}\langle \phi \left(m\left(t\right)+\tilde{h}\left(t\right)\right)\phi \left(m\left(s\right)+\tilde{h}\left(s\right)\right)\rangle \;. \end{array}$$ (10)

Here and in the following, angular brackets denote expectation values over the distribution of the stochastic process $\tilde{h}\left(t\right)$, which approximates population averages in the full network. The mean-field estimate for the mean component *m*(*t*) of *h*_*i*_ therefore evolves according to Eq 2 with $\nu \left(t\right)=\langle \phi \left(m\left(t\right)+\tilde{h}\left(t\right)\right)\rangle$, the mean-field estimate of the mean population firing rate.

We derive coupled equations for the time evolution of the two-time autocorrelation function $c\left(t,s\right)=\langle \tilde{h}\left(t\right)\tilde{h}\left(s\right)\rangle$, which explicitly depends on the two times *t* and *s*. Taking the temporal derivative of *c*(*t*, *s*) with respect to *s* and using Eq 9, we obtain

$$\begin{array}{c} \tau \frac{d}{ds}c\left(t,s\right)=-c\left(t,s\right)+r\left(t,s\right), \end{array}$$ (11)

where $r\left(t,s\right)=\langle \tilde{h}\left(t\right)\eta \left(s\right)\rangle$, which we take as an auxiliary function. Taking the temporal derivative of *r*(*t*, *s*) with respect to *t* we arrive at an expression for the time evolution of the function *r*(*t*, *s*):

$$\begin{array}{c} \tau \frac{d}{dt}r\left(t,s\right)=-r\left(t,s\right)+q\left(t,s\right)\;, \end{array}$$ (12)

where $q\left(t,s\right)=g^{2}\langle \phi \left(m\left(t\right)+\tilde{h}\left(t\right)\right)\phi \left(m\left(s\right)+\tilde{h}\left(s\right)\right)\rangle$ (see Eq 10). The idea of considering an auxiliary function *r* has been proposed for a discrete-time model previously [34]. Together, the dynamic mean-field equations for *m*(*t*), *c*(*t*, *s*) and *r*(*t*, *s*) form a closed system of self-consistent dynamic equations and can be solved forward in time, both in *s* and *t*, by integrating them on a two-dimensional grid from some initial condition for *m*, *c* and *r*. The integration requires *q*(*t*, *s*), which can be calculated by evaluating a Gaussian double integral that depends on *c*(*t*, *s*), *c*(*t*, *t*), *c*(*s*, *s*), *m*(*t*) and *m*(*s*). For the threshold-linear transfer function *ϕ*(*x*) = max(*x*, 0), one integral can be evaluated analytically, which allows for an efficient numerical implementation using adaptive Gauss–Kronrod integration [35–37]. The non-stationary DMFT accurately captures the time-dependent mean population rate *ν*(*t*) and the two-time autocorrelation function (Fig 8) both in the (cyclostationary) chaotic and in the (periodic) driven stable regime.

To quantify chaos, we calculate the largest Lyapunov exponent using DMFT by considering the distance between the states of two replicas of the system with identical realization of the network couplings *J*_*ij*_, identical external input *δI*_*i*_(*t*), but different initial conditions [4, 14, 38]. The squared distance between the two systems can be expressed in terms of their two-time autocorrelations *c*^11^, *c*^22^, and the cross-correlations *c*^12^, *c*^21^ between them [4],

$$\begin{array}{c} d\left(t,s\right)=c^{11}\left(t,s\right)+c^{22}\left(t,s\right)-c^{12}\left(t,s\right)-c^{21}\left(t,s\right)\;, \end{array}$$ (13)

with *c*^21^(*t*, *s*) = *c*^12^(*s*, *t*). We next linearize the dynamics of the cross-correlation function and thereby of the squared distance around the solution that is perfectly correlated between the two replicas: *c*^12^(*t*, *s*) = *c*(*t*, *s*) + *ϵ k*(*t*, *s*), *ϵ* ≪ 1. This yields a linear partial differential equation for the temporal evolution of the squared distance *d*(*t*) between infinitesimal perturbations [4]:

$$\begin{array}{c} (\tau \partial_{t}+1)(\tau \partial_{s}+1)k\left(t,s\right)=q_{\phi^{'}\phi^{'}}\left(t,s\right)k\left(t,s\right)\;, \end{array}$$ (14)

with *d*(*t*) = −2*ϵ k*(*t*, *t*) and $q_{\phi^{'}\phi^{'}}\left(t,s\right)=g^{2}\langle \phi^{'}\left(m\left(t\right)+\tilde{h}\left(t\right)\right)\phi^{'}\left(m\left(s\right)+\tilde{h}\left(s\right)\right)\rangle$.

In contrast to earlier approaches [1, 4, 16], where the statistics were stationary, for common input, the two-time autocorrelation function is required to evaluate Eq 14, which makes *q*_*ϕ*^′^*ϕ*^′^_(*t*, *s*) explicitly dependent on *t* and *s* and not only on the difference *t* − *s*. Eq 14 can be solved by integrating forward on a two-dimensional grid, similarly to the solution of the two-time autocorrelation function.

Specifically, similar to the case of the equations for *c* and *r*, we solve Eq 14 by rewriting it as two differential equations for *k* and an auxiliary variable *l*,

$$\begin{array}{c} \tau \frac{d}{ds}k\left(t,s\right)=-k\left(t,s\right)+l\left(t,s\right)\;, \end{array}$$ (15)

and

$$\begin{array}{c} \tau \frac{d}{dt}l\left(t,s\right)=-l\left(t,s\right)+k\left(t,s\right)q_{\phi^{'}\phi^{'}}\left(t,s\right)\;. \end{array}$$ (16)

The function *q*_*ϕ*^′^*ϕ*^′^_(*t*, *s*) can be calculated by evaluating a Gaussian double integral that depends on *c*(*t*, *s*), *c*(*t*, *t*), *c*(*s*, *s*), *m*(*t*) and *m*(*s*), which we obtained above (Eqs 10–12).

The largest Lyapunov exponent is given by the the average exponential growth rate of *k*(*t*, *t*), discarding an initial transient:

$$\begin{array}{c} \lambda_{1}=\lim_{t\to \infty }\frac{1}{2t}\log\frac{\left|k(t,t)\right|}{\left|k(0,0)\right|}. \end{array}$$ (17)

Example code in Julia 1.8 for solving the non-stationary DMFT and calculating autocorrelations and the largest Lyapunov exponent is available at github.com/RainerEngelken/NonstationaryDynamicMeanFieldTheory.

#### Quasi-static approximation for low-frequency input

We consider very slow common input modulations with *τ f* ≪ 1. In this case, the network can be approximately described by stationary DMFT which, for $g>g_{\text{crit}}=\sqrt{2}$, yields chaotic dynamics for any constant positive external input [16]. However, when $\delta I\left(t\right)<-\sqrt{N}I_{0}$, neurons are driven by negative input and the network becomes silent. During these silent episodes, because of the dissipation coming from the leak of the individual neurons, the dynamics is transiently very stable. In other words, for silenced networks, the largest Lyapunov exponent is $\lambda_{1}^{\text{local}}=-1/\tau$ as the Jacobian matrix of the dynamics is $-\frac{1}{\tau }\delta_{ij}$.

The critical input amplitude $I_{1}^{\text{crit}}$ occurs when these silent episodes on average compensate transiently chaotic episodes. Since the Lyapunov exponent of the chaotic episodes is small for *g* close to $g_{\text{crit}}$, very short silent episodes suffice to suppress chaos. Therefore, the critical input amplitude in the limit of small $g-g_{\text{crit}}$ in the quasi-static approximation is expected to be

$$\begin{array}{c} I_{1}^{\text{crit}}=\sqrt{N}I_{0}. \end{array}$$ (18)

For increasing *g*, the positive input episodes become locally more chaotic, which increases $I_{1}^{\text{crit}}$. Thus, in the quasi-static approximation, the largest Lyapunov exponent depends on *g* and the distribution of the time-varying input

$$\begin{array}{ccc} \lambda_{1}\left(g,\;I_{1}\right) & = & \lim_{T\to \infty }\frac{1}{T}\int_{0}^{T}\lambda_{1}^{\text{local}}\left(g,\sqrt{N}I_{0}+\delta I\left(t\right)\right)\;dt \end{array}$$ (19)

$$\begin{array}{cc} = & -\frac{1}{\tau }\int_{-\infty }^{0}p\left(I\right)\;dI+\lambda_{1}^{\text{const}}\left(g\right)\int_{0}^{\infty }p\left(I\right)\;dI\;, \end{array}$$ (20)

where $\lambda_{1}^{\text{const}}\left(g\right)$ is the largest Lyapunov exponent for constant input [16], and *I* is integrated over the probability distribution of $\delta I\left(t\right)+\sqrt{N}I_{0}$. In the second equality, we used the fact that, for constant positive external input, the Lyapunov exponent is independent of the input value *I* for threshold-linear transfer function due to its positive homogeneity. For *δI*(*t*) = *I*_1_ sin(2*πft*), Eq 20 becomes

$$\begin{array}{ccc} \lambda_{1}\left(g,I_{1}\right) & \approx & -\frac{1}{\tau }\arccos(\frac{\sqrt{N}I_{0}}{I_{1}})+\lambda_{1}^{\text{const}}\left(g\right)(1-\arccos(\frac{\sqrt{N}I_{0}}{I_{1}}))\;. \end{array}$$ (21)

Solving λ_1_(*g*, *I*_1_) = 0 for *I*_1_ yields

$$\begin{array}{ccc} I_{1}^{\text{crit}}\left(g\right) & \approx & \sqrt{N}I_{0}\sec(\frac{\pi \lambda_{1}^{\text{const}}\left(g\right)}{1/\tau +\lambda_{1}^{\text{const}}\left(g\right)})\;. \end{array}$$ (22)

$\lambda_{1}^{}$ is calculated analytically using stationary DMFT [15, 16]. This is the quasi-static approximation plotted as dotted lines in Figs 3C and 5. Note that $I_{1}^{\text{crit}}$ diverges when *g* is so large that $\lambda_{1}^{\text{const}}=1/\tau$. For larger *g*, arbitrary strong slow inputs cannot suppress chaos. For *g* close to the autonomous transition $g_{\text{crit}}$, we can use the analytical approximation $\lambda_{1}^{\text{const}}(g)=c(g-g_{\text{crit}})$[16], where *c* is a constant of order 1. Thus,

$$\begin{array}{ccc} I_{1}^{\text{crit}}\left(g\right) & \approx & \sqrt{N}I_{0}(1+\frac{\pi^{2}c^{2}}{2}{\left(g-g_{\text{crit}}\right)}^{2})\;. \end{array}$$ (23)

In the case of time-varying common input modulations generated by an OU process $\frac{d}{dt}\delta I\left(t\right)=-\frac{1}{\tau_{s}}\delta I\left(t\right)+\sqrt{2D}\xi \left(t\right)$, where *ξ*(*t*) is Gaussian white noise with zero mean and unit variance, a similar calculation based on $p\left(I\right)=\frac{1}{\sqrt{2\pi \tau_{s}D}}\;e^{-\frac{{\left(I-\sqrt{N}I_{0}\right)}^{2}}{2\tau_{s}D}}$ leads to

$$\begin{array}{ccc} \lambda_{1}\left(g,\;D\right) & \approx & -\frac{1}{2\tau }\mathrm{erfc}(\frac{\sqrt{N}I_{0}}{\sqrt{2\tau_{s}D}})+\lambda_{1}^{\text{const}}\left(g\right)(1-\frac{\mathrm{erfc}(\frac{\sqrt{N}I_{0}}{\sqrt{2\tau_{s}D}})}{2}). \end{array}$$ (24)

Solving $\lambda_{1}\left(g,\;D\right)\overset{!}{=}0$ by *D* determines the critical input amplitude

$$\begin{array}{ccc} D^{\text{crit}}\left(g\right) & \approx & \frac{NI_{0}^{\;2}}{2\tau_{s}{\left[{\mathrm{erfc}}^{-1}(\frac{2\lambda_{1}^{\text{const}}\left(g\right)}{\frac{1}{\tau }+\lambda_{1}^{\text{const}}\left(g\right)})\right]}^{2}}. \end{array}$$ (25)

Again, when *g* is sufficiently large such that the largest Lyapunov exponent during the chaotic episodes reaches $\lambda_{1}^{\text{const}}=\frac{1}{\tau }$, OU-input of any amplitude cannot suppress chaos.

#### High-frequency limit

For high input frequencies, the leak term in the network dynamics acts as a low-pass filter of the external input effectively attenuating it by a factor of $1/\sqrt{1+{\left(2\pi f\tau \right)}^{2}}$. Thus, common input is low-pass filtered in Eq 2a. Analyzing the attenuation in Eq 2a, we find a linear dependence for high input frequencies,

$$\begin{array}{c} I_{1}^{\text{crit}}\left(f\right)\propto \tau f\;. \end{array}$$ (26)

The expected high-frequency scaling is visible in Fig 5A. The crossover to the linear scaling regime of $I_{1}^{\text{crit}}$ occurs at $f_{c}\propto \frac{\sqrt{N}I_{0}}{\tau }.$ We observed such a behavior of the crossover also in numerical simulations.

### Independent input

#### Stationary dynamic mean-field theory

In the case of independent input, we obtain stationary autocorrelations and constant mean currents self-consistently similar to [3], but additionally also taking a mean component *m* into account [15, 16]. Moreover, we obtain the largest Lyapunov exponent in a similar way to previous dynamic mean-field approaches [1, 4, 16]. The stationary DMFT accurately captures the constant mean population rate *ν* and the stationary autocorrelation function obtained from numerical simulations (Fig 9), both in the chaotic and in the driven stable regime.

#### Low-frequency limit

In the low-frequency limit, suppression of chaos by independent input can be understand rather intuitively. The network receives quenched independent (heterogeneous) input, which widens the distribution of $\tilde{h}$ and reduces the spectral radius of the Jacobian of the dynamics and thus results in a suppression of chaos [2, 4]. At values of *g* close to the transition to chaos $g_{\text{crit}}=\sqrt{2}$, only a very small input amplitude *I*_1_ of the quenched input is necessary to suppress chaos. We find that in the low-frequency limit

$$\begin{array}{c} I_{1}^{\text{crit}}\left(g\right)=2\sqrt[{4}]{2}\sqrt{\pi }\frac{I_{0}}{J_{0}}\sqrt{g-g_{\text{crit}}}\;. \end{array}$$ (27)

Thus, close to the transition to chaos, arbitrary small *I*_1_ can suppress chaos in the independent case, while for common input in this limit, $I_{1}^{\text{crit}}=\sqrt{N}I_{0}$ as we have discussed above. This is consistent with the results in Fig 3C.

#### High-frequency limit

Similar to common input, for high frequencies, the leak term in the Eq 4b for ${\tilde{h}}_{i}$ attenuates the effective input amplitude by a factor of $1/\sqrt{1+{\left(2\pi f\tau \right)}^{2}}$. Thus, in the high-frequency limit we expect the same linear scaling as in the common input case,

$$\begin{array}{c} I_{1}^{\text{crit}}\left(f\right)\propto \tau f. \end{array}$$ (28)

Unlike the common input case, the crossover to this scaling is not expected to depend on network size for large *N*, as the suppression of chaos is not impaired by the cancellation of the external input by recurrent feedback. This scaling is observed in Fig 5A.

#### Common vs independent input in networks with zero mean coupling and transfer function *ϕ*(*x*) = tanh(*x*)

**Fig 10:**
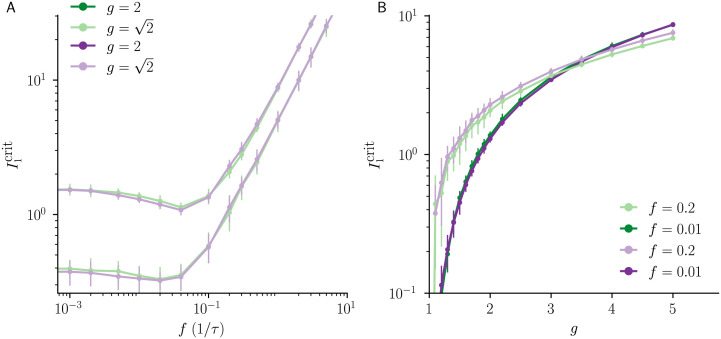
No qualitative difference in chaos suppression by common vs independent input in canonical random networks. **A)** $I_{1}^{crit}$ as a function of input frequency *f* ($g=\sqrt{2}$ light color, *g* = 2 dark color). $I_{1}^{\text{crit}}$ has a minimum for both common and independent input. The independent input case is identical to the scenario studied in [3]. At high *f*, the low-pass filter effect of the leak term attenuates the external input for both cases, thus resulting in a linearly increasing $I_{1}^{\text{crit}}$. **B)** Dependence of $I_{1}^{\text{crit}}$ on the gain parameter *g* for both low input frequency (*f* = 0.01/*τ*, dark color) and high input frequency (*f* = 0.2/*τ*, light color), showing a monotonic increase. Error bars indicate ±2 std. Model parameters: *N* = 5000, $g\in \{\sqrt{2},2$}, *f* ∈ {0.01, 0.2}/*τ*, *I*_0_ = *J*_0_ = 0.

For completeness, we also numerically studied suppression of chaos by common vs independent input in standard non-balanced networks with zero mean coupling, zero mean input, and transfer function *ϕ*(*x*) = tanh(*x*) [1]. We considered, as in [3]

$$\begin{array}{c} \tau \frac{dh_{i}}{dt}=-h_{i}+\sum_{j=1}^{N}J_{ij}\phi (h_{j})+\delta I_{i}\left(t\right)\;, \end{array}$$ (29)

with i.i.d. Gaussian-distributed random couplings $J_{ij}∼N\left(0,g^{2}/N\right)$, where the gain parameter *g* controls the weight heterogeneity of the network. We use an identical definition of the time-varying common and independent input, as before in Eq 1. The independent input case is identical to the scenario studied in [3]. As expected, we found that in contrast to balanced networks, there is no qualitative difference between $I_{1}^{\text{crit}}$ for common versus independent input (Fig 10). To our surprise, the difference is small even quantitatively. This suggests that for the standard, non-balanced networks, the results obtained by considering independently time-varying inputs [2–4] may also carry over to correlated inputs.

#### Quantification of chaos

Chaotic systems are sensitive to initial conditions, and almost all infinitesimal perturbations *ϵ**u***_0_ of the initial condition ***h***_0_ + *ϵ**u***_0_ grow asymptotically exponentially ||*ϵ**u***_*t*_|| ≈ exp(λ_1_*t*)||*ϵ**u***_0_||. The largest Lyapunov exponent λ_1_ measures the average rate of exponential divergence or convergence of nearby initial conditions,

$$\begin{array}{c} \lambda_{1}\left(h_{0}\right)=\lim_{t\to \infty }\frac{1}{t}\lim_{\epsilon \to 0}\log\frac{||\epsilon u_{t}\left|\right|}{||\epsilon u_{0}\left|\right|}\;. \end{array}$$ (30)

For dynamics on an ergodic attractor, λ_1_ does not depend on the initial condition **h**_0_. We calculated the largest Lyapunov exponent of the networks dynamics in two different ways, both based on analytical expressions of the Jacobian of the dynamics [17, 39] and with direct numerical simulations tracking the distance between two nearby trajectories. Based on the Lyapunov exponent, we determine the critical input amplitude $I_{1}^{\text{crit}}$ using a bisection method with a relative precision of one percent.

#### Target-based learning

We employ a recently developed target-based learning algorithm called full-FORCE [6, 8]. The learning procedure is the following: a student network (S) learns a task by matching its total incoming currents $\eta_{i}^{S}(t)=\sum_{j}\;J_{ij}^{S}\phi (h_{j}^{S}(t))+\sqrt{N}I_{0}$ to those of a randomly coupled teacher network (T), that is driven by the desired output signal, i.e. $\eta_{i}^{T}(t)=\sum_{j}\;J_{ij}^{T}\phi (h_{j}^{T}(t))+\sqrt{N}I_{0}+\delta I_{i}(t)$; in the special case of a sinusoidal signal $F^{\text{out}}(t)=\sin\left(2\pi ft\right)$, with *δI*_*i*_(*t*) = *I*_1_sin(2*πft* + *θ*_*i*_) (as in the main text). The synaptic matrix $J_{ij}^{S}$ is trained using an online procedure so that the student network can generate the target output autonomously, $z\left(t\right)=\sum_{i}\;w_{i}\;\phi (h_{i}^{S}\left(t\right))$, where *z*(*t*) is a linear readout of the student network’s firing rates. Both the recurrent couplings $J_{ij}^{S}$ and the readout weights *w*_*i*_ are trained to produce the prescribed output signal, i.e., $z\left(t\right)\approx F^{\text{out}}\left(t\right)$.

The incoming currents in the teacher and student network are matched via an online minimization of the following error function for each neuron *i* ∈ 1, …*N*,

$$\begin{array}{c} L_{i}=\int_{0}^{T_{tot}}dt{\left(\eta_{i}^{T}\;(t)-\sum_{j=1}^{N}J_{ij}^{S}\phi (h_{j}^{S}\left(t\right))-\sqrt{N}I_{0}\right)}^{2}+\beta \sum_{j}{\left(J_{ij}^{S}\right)}^{2}, \end{array}$$ (31)

Following [5, 6], recursive least square (RLS) is used to minimize the training error (Eq 31) and to concurrently learn the readout weight vector *w*_*i*_. We used a balanced initialization for both the teacher and student network: $J_{ij}^{T}$ and $J_{ij}^{S}$ are independently initialized as i.i.d. Gaussian matrices with mean $-J_{0}/\sqrt{N}$ and variance *g*^2^/*N*. Both networks receive a constant external input $\sqrt{N}I_{0}$. Euler integration was used with a time step of Δ*t* = 0.01. The regularization parameter for RLS was *β* = 1.

Test error is computed over a testing period $T_{\text{test}}=50T_{osc}$, which we take as 50 periods of the desired output signal, i.e. $T_{\text{test}}=50/f$, as

$$\begin{array}{c} E_{\text{test}}=\frac{\int_{0}^{T_{\text{test}}}dt({\left(z(t)-F^{\text{out}}(t)\right)}^{2}}{\int_{0}^{T_{\text{test}}}dt{\left(F^{\text{out}}(t)\right)}^{2}}\;. \end{array}$$ (32)

For a periodic target $F^{\text{out}}$, testing is interleaved with training so that the network state $h_{i}^{S}\left(t\right)$ is usually close to the target trajectory. In this case, a sufficiently low test error usually implies the presence of a stable limit cycle, and the periodic output is reproduced, up to a phase shift, starting from any initial condition.

## References

[1] Sompolinsky H, Crisanti A, Sommers HJ. Chaos in Random Neural Networks. Physical Review Letters. 1988;61(3):259–262. doi: 10.1103/PhysRevLett.61.259 10039285

[2] Molgedey L, Schuchhardt J, Schuster HG. Suppressing chaos in neural networks by noise. Physical Review Letters. 1992;69(26):3717–3719. doi: 10.1103/PhysRevLett.69.3717 10046895

[3] Rajan K, Abbott LF, Sompolinsky H. Stimulus-dependent suppression of chaos in recurrent neural networks. Physical Review E. 2010;82(1):011903. doi: 10.1103/PhysRevE.82.011903 20866644 PMC10683875

[4] Schuecker J, Goedeke S, Helias M. Optimal Sequence Memory in Driven Random Networks. Physical Review X. 2018;8(4):041029. doi: 10.1103/PhysRevX.8.041029

[5] Sussillo D, Abbott LF. Generating Coherent Patterns of Activity from Chaotic Neural Networks. Neuron. 2009;63(4):544–557. doi: 10.1016/j.neuron.2009.07.018 19709635 PMC2756108

[6] DePasquale B, Cueva CJ, Rajan K, Escola GS, Abbott LF. full-FORCE: A target-based method for training recurrent networks. PLOS ONE. 2018;13(2):e0191527. doi: 10.1371/journal.pone.0191527 29415041 PMC5802861

[7] Kim CM, Chow CC. Learning recurrent dynamics in spiking networks. eLife. 2018;7:e37124. doi: 10.7554/eLife.37124 30234488 PMC6195349

[8] Ingrosso A, Abbott LF. Training dynamically balanced excitatory-inhibitory networks. PLOS ONE. 2019;14(8):e0220547. doi: 10.1371/journal.pone.0220547 31393909 PMC6687153

[9] Ozeki H, Finn IM, Schaffer ES, Miller KD, Ferster D. Inhibitory Stabilization of the Cortical Network Underlies Visual Surround Suppression. Neuron. 2009;62(4):578–592. doi: 10.1016/j.neuron.2009.03.028 19477158 PMC2691725

[10] Ahmadian Y, Rubin DB, Miller KD. Analysis of the stabilized supralinear network. Neural computation. 2013;25(8):1994–2037. doi: 10.1162/NECO_a_00472 23663149 PMC4026108

[11] Wolf F, Engelken R, Puelma-Touzel M, Weidinger JDF, Neef A. Dynamical models of cortical circuits. Current Opinion in Neurobiology. 2014;25:228–236. doi: 10.1016/j.conb.2014.01.017 24658059

[12] Sanzeni A, Akitake B, Goldbach HC, Leedy CE, Brunel N, Histed MH. Inhibition stabilization is a widespread property of cortical networks. eLife. 2020;9:e54875. doi: 10.7554/eLife.54875 32598278 PMC7324160

[13] van Vreeswijk C, Sompolinsky H. Chaos in Neuronal Networks with Balanced Excitatory and Inhibitory Activity. Science. 1996;274(5293):1724–1726. doi: 10.1126/science.274.5293.1724 8939866

[14] van Vreeswijk C, Sompolinsky H. Chaotic Balanced State in a Model of Cortical Circuits. Neural Computation. 1998;10(6):1321–1371. doi: 10.1162/089976698300017214 9698348

[15] Harish O, Hansel D. Asynchronous Rate Chaos in Spiking Neuronal Circuits. PLoS Comput Biol. 2015;11(7):e1004266. doi: 10.1371/journal.pcbi.1004266 26230679 PMC4521798

[16] Kadmon J, Sompolinsky H. Transition to Chaos in Random Neuronal Networks. Physical Review X. 2015;5(4):041030. doi: 10.1103/PhysRevX.5.041030

[17] Engelken R, Wolf F, Abbott LF. Lyapunov spectra of chaotic recurrent neural networks. arXiv:200602427 [nlin, q-bio]. 2020;.

[18] Kadmon J, Timcheck J, Ganguli S. Predictive coding in balanced neural networks with noise, chaos and delays. Advances in neural information processing systems. 2020;33.

[19] Ahmadian Y, Miller KD. What is the dynamical regime of cerebral cortex? Neuron. 2021;109(21):3373–3391. doi: 10.1016/j.neuron.2021.07.031 34464597 PMC9129095

[20] Mastrogiuseppe F, Ostojic S. Intrinsically-generated fluctuating activity in excitatory-inhibitory networks. PLOS Computational Biology. 2017;13(4):e1005498. doi: 10.1371/journal.pcbi.1005498 28437436 PMC5421821

[21] Khajeh R, Fumarola F, Abbott LF. Sparse balance: Excitatory-inhibitory networks with small bias currents and broadly distributed synaptic weights. PLOS Computational Biology. 2022;18(2):e1008836. doi: 10.1371/journal.pcbi.1008836 35139071 PMC8827417

[22] Renart A, Rocha Jdl, Bartho P, Hollender L, Parga N, Reyes A, et al. The Asynchronous State in Cortical Circuits. Science. 2010;327(5965):587–590. doi: 10.1126/science.1179850 20110507 PMC2861483

[23] Darshan R, Wood WE, Peters S, Leblois A, Hansel D. A canonical neural mechanism for behavioral variability. Nature Communications. 2017;8:15415. doi: 10.1038/ncomms15415 28530225 PMC5458148

[24] Tetzlaff T, Helias M, Einevoll GT, Diesmann M. Decorrelation of Neural-Network Activity by Inhibitory Feedback. PLOS Comput Biol. 2012;8(8):e1002596. doi: 10.1371/journal.pcbi.1002596 23133368 PMC3487539

[25] Rosenbaum R, Smith MA, Kohn A, Rubin JE, Doiron B. The spatial structure of correlated neuronal variability. Nature neuroscience. 2017;20(1):107–114. doi: 10.1038/nn.4433 27798630 PMC5191923

[26] Aljadeff J, Stern M, Sharpee T. Transition to Chaos in Random Networks with Cell-Type-Specific Connectivity. Physical Review Letters. 2015;114(8):088101. doi: 10.1103/PhysRevLett.114.088101 25768781 PMC4527561

[27] Aljadeff J, Renfrew D, Vegué M, Sharpee TO. Low-dimensional dynamics of structured random networks. Physical Review E. 2016;93(2):022302. doi: 10.1103/PhysRevE.93.022302 26986347 PMC4820296

[28] Mastrogiuseppe F, Ostojic S. Linking Connectivity, Dynamics, and Computations in Low-Rank Recurrent Neural Networks. Neuron. 2018;99(3):609–623.e29. doi: 10.1016/j.neuron.2018.07.003 30057201

[29] Landau ID, Sompolinsky H. Macroscopic fluctuations emerge in balanced networks with incomplete recurrent alignment. Physical Review Research. 2021;3(2):023171. doi: 10.1103/PhysRevResearch.3.023171

[31] Perich MG, Arlt C, Soares S, Young ME, Mosher CP, Minxha J, et al. Inferring brain-wide interactions using data-constrained recurrent neural network models. 2021; p. 2020.12.18.423348. 10.1016/j.neuron.2026.07.016 PMC13565020 42567158

[30] Pandarinath C, O’Shea DJ, Collins J, Jozefowicz R, Stavisky SD, Kao JC, et al. Inferring single-trial neural population dynamics using sequential auto-encoders. Nature Methods. 2018;15(10):805–815. doi: 10.1038/s41592-018-0109-9 30224673 PMC6380887

[32] Stern M, Sompolinsky H, Abbott LF. Dynamics of random neural networks with bistable units. Physical Review E. 2014;90(6):062710. doi: 10.1103/PhysRevE.90.062710 25615132 PMC4348075

[33] Muscinelli SP, Gerstner W, Schwalger T. How single neuron properties shape chaotic dynamics and signal transmission in random neural networks. PLOS Computational Biology. 2019;15(6):e1007122. doi: 10.1371/journal.pcbi.1007122 31181063 PMC6586367

[34] Wainrib G, Galtier MN. A local Echo State Property through the largest Lyapunov exponent. Neural Networks. 2016;76:39–45. doi: 10.1016/j.neunet.2015.12.013 26849424

[35] Kronrod AS. Integration with Control of Accuracy. Soviet Physics Doklady. 1964;9:17.

[36] Johnson SG. QuadGK.jl: Gauss–Kronrod integration in Julia; 2013. Available from: https://github.com/JuliaMath/QuadGK.jl.

[37] Example code in Julia 1.8 for solving the non-stationary DMFT and calculating autocorrelations and the largest Lyapunov exponent is available at github.com/RainerEngelken/NonstationaryDynamicMeanFieldTheory.

[38] Derrida B, Pomeau Y. Random Networks of Automata: A Simple Annealed Approximation. Europhysics Letters (EPL). 1986;1(2):45–49. doi: 10.1209/0295-5075/1/2/001

[39] Benettin G, Galgani L, Giorgilli A, Strelcyn JM. Lyapunov Characteristic Exponents for smooth dynamical systems and for hamiltonian systems; A method for computing all of them. Part 2: Numerical application. Meccanica. 1980;15(1):21–30. doi: 10.1007/BF02128236

